# Correction: Hypoxemia prediction in pediatric patients under general anesthesia using machine learning: A retrospective observational study and external validation

**DOI:** 10.1371/journal.pone.0355945

**Published:** 2026-08-11

**Authors:** Sujin Baek, Jung-Bin Park, Jihye Heo, Kyungsang Kim, Donghyeon Baek, Chahyun Oh, Hyung-Chul Lee, Dongheon Lee, Boohwi Hong

The ORCID iD is missing for the third author. Please see the author’s ORCID iD here:

- Author Jihye Heo’s ORCID iD is: 0009-0000-8415-0230 (https://orcid.org/0009-0000-8415-0230).
