# Supplementary material for: Hypoxemia prediction in pediatric patients under general anesthesia using machine learning: A retrospective observational study and external validation
Source: PLoS One. 2026 Jan 8;21(1):e0339276. doi: 10.1371/journal.pone.0339276 (PMC12782441; doi:10.1371/journal.pone.0339276)
Supplement: S2 Table — This table presents the distribution of labeled segments across the training, internal validation, and external validation datasets, highlighting that hypoxemia-labeled segments were more prevalent in the CNUH dataset (1.42%) than in the SNUH datasets (1.10% in training and 1.08% in internal validation). Abbreviations: SNUH, Seoul National University Hospital; CNUH, Chungnam National University Hospital. (DOCX) [file pone.0339276.s002.docx]

**S2 Table. Counts of each labeled segment in the dataset.** This table presents the distribution of labeled segments across the training, internal validation, and external validation datasets, highlighting that hypoxemia-labeled segments were more prevalent in the CNUH dataset (1.42%) than in the SNUH datasets (1.10% in training and 1.08% in internal validation).

| **Label** | **Training dataset (SNUH)** | **Internal validation dataset (SNUH)** | **External validation dataset (CNUH)** |
| --- | --- | --- | --- |
| 0 (normal) | 4,793,665 | 578,858 | 497,311 |
| 1 (hypoxemia) | 53,539 | 6,316 | 7,610 |
| Total | 4,847,204 | 585,174 | 504,921 |
| Proportion of label 1 (hypoxemia) segment(%) | 1.10 | 1.08 | 1.42 |

Abbreviations: SNUH, Seoul National University Hospital; CNUH, Chungnam National University Hospital.
