# Supplementary material for: Hypoxemia prediction in pediatric patients under general anesthesia using machine learning: A retrospective observational study and external validation
Source: PLoS One. 2026 Jan 8;21(1):e0339276. doi: 10.1371/journal.pone.0339276 (PMC12782441; doi:10.1371/journal.pone.0339276)
Supplement: S3 Table — (DOCX) [file pone.0339276.s003.docx]

**S3 Table. Hyperparameter search space and final selected values for the machine learning models.**

| **Model** | **Parameter** | **Search Space** | **Final Value** |
| --- | --- | --- | --- |
| LSTM | Llayer (Number of LSTM layers) | {1, 2} | 1 |
|  | Lnode (LSTM hidden units) | {16, 32, 64} | 64 |
|  | Fnode (Final dense layer units) | {16, 32, 64, 128} | 16 |
|  | dropout (Dropout rate) | {0.2, 0.5} | 0.5 |
| XGBoost | learning_rate | {0.02, 0.04, 0.08} | 0.04 |
|  | max_depth | {3, 4, 5} | 5 |
|  | min_child_weight | {1, 2, 4} | 2 |
|  | gamma | {0, 0.2, 0.4} | 0.4 |
|  | subsample | {0.5, 0.8} | 0.5 |
|  | colsample_bytree | {0.5, 0.8} | 0.5 |
| Transformer | Nfilt (Number of filters in convolution) | {16, 32, 64} | 64 |
|  | Nhead (Number of attention heads) | {2, 3, 4} | 3 |
|  | Kdim (key_dim) (Size of each attention head for Key and Query) | {16, 32, 64} | 32 |
|  | Fnode (Nodes in final dense layer) | {32, 64, 128} | 32 |
|  | Clayer (Number of convolutional layers) | {1, 2, 3, 4} | 1 |
|  | Tlayer (Number of transformer layers) | {1, 2, 3} | 3 |
|  | Filtsize (Filter size in convolution) | {5, 7, 9, 11} | 5 |
|  | Poolsize (Pooling size) | {2, 4, 8} | 4 |
| InceptionTime | Nb_filters | Adopted from original paper | 16 |
|  | Bottle_size | Adopted from original paper | 32 |
|  | Depth | Adopted from original paper | 6 |
|  | Kernel_size | Adopted from original paper | 20 |
| **Training Parameters** | | | |
| n_splits | Number of folds for cross-validation | - | 5 |
| init_lr | Initial learning rate | - | 1.0e-3 |
| num_epochs | Number of training epochs | - | 100 |
| batch_size | Batch size for training | - | 1024 |
| window_size | Observation window length (time points) | - | 30 |

Abbreviations: LSTM, long short-term memory
