# Supplementary material for: Hypoxemia prediction in pediatric patients under general anesthesia using machine learning: A retrospective observational study and external validation
Source: PLoS One. 2026 Jan 8;21(1):e0339276. doi: 10.1371/journal.pone.0339276 (PMC12782441; doi:10.1371/journal.pone.0339276)
Supplement: S4 Table — The performance of the XGBoost and Transformer models for predicting hypoxemia in pediatric patients under general anesthesia was compared before and after normalization. The performance metrics, including the AUROC, AUPRC, and F1 score, were evaluated on both internal and external validation datasets. The highest values for each metric in both datasets are highlighted in bold. Abbreviations: AUROC, area under the receiver operating characteristic curve; AUPRC, area under the precision-recall curve; w/o, without; w/, with; norm., normalization. (DOCX) [file pone.0339276.s004.docx]

S4 Table. Comparative performance of machine learning models for hypoxemia prediction in pediatric patients after normalization. The performance of the XGBoost and Transformer models for predicting hypoxemia in pediatric patients under general anesthesia was compared before and after normalization. The performance metrics, including the AUROC, AUPRC, and F1 score, were evaluated on both internal and external validation datasets. The highest values for each metric in both datasets are highlighted in bold.

|  | | Internal validation | | | External validation | | |
| --- | --- | --- | --- | --- | --- | --- | --- |
|  |  | AURO | AUPRC | F1 score | AURO | AUPRC | F1 score |
| XGBoost | w/o norm. | **0.8550** | **0.1816** | **0.2382** | 0.7857 | 0.0402 | 0.0824 |
|  | w/ norm. | 0.8044 | 0.1385 | 0.2400 | 0.7075 | 0.0342 | 0.0817 |
| Transformer | w/o norm. | 0.7934 | 0.0505 | 0.1283 | **0.8501** | **0.0595** | **0.1227** |
|  | w/ norm. | 0.8094 | 0.0794 | 0.1642 | 0.7111 | 0.0406 | 0.1002 |

Abbreviations: AUROC, area under the receiver operating characteristic curve; AUPRC, area under the precision-recall curve; w/o, without; w/, with; norm., normalization.
