# Supplementary material for: Hypoxemia prediction in pediatric patients under general anesthesia using machine learning: A retrospective observational study and external validation
Source: PLoS One. 2026 Jan 8;21(1):e0339276. doi: 10.1371/journal.pone.0339276 (PMC12782441; doi:10.1371/journal.pone.0339276)
Supplement: S5 Table — The performance of the XGBoost and Transformer models for hypoxemia prediction in pediatric patients under general anesthesia, stratified by age subgroups, shows variations in the AUROC, AUPRC, and F1 scores across internal and external validation datasets. Abbreviations: AUROC, area under the receiver operating characteristic curve; AUPRC, area under the precision-recall curve. (DOCX) [file pone.0339276.s005.docx]

**S5 Table.** **Comparative performance of machine learning models for hypoxemia prediction in pediatric patients after training by age subgroup.** The performance of the XGBoost and Transformer models for hypoxemia prediction in pediatric patients under general anesthesia, stratified by age subgroups, shows variations in the AUROC, AUPRC, and F1 scores across internal and external validation datasets.

|  | Age subgroup | Internal validation | | | External validation | | |
| --- | --- | --- | --- | --- | --- | --- | --- |
|  |  | AUROC | AUPRC | F1 score | AUROC | AUPRC | F1 score |
| XGBoost | All | 0.8550 | 0.1816 | 0.2382 | 0.7857 | 0.0402 | 0.0824 |
|  | 0–2 yr | **0.8760** | **0.1690** | **0.2671** | 0.7357 | 0.0308 | 0.0677 |
|  | 2–8 yr | 0.8438 | 0.1092 | 0.1827 | **0.7889** | **0.0368** | **0.0844** |
|  | 8–18 yr | 0.7981 | 0.1347 | 0.1999 | 0.7865 | 0.0353 | 0.0711 |
| Transformer | All | 0.7934 | 0.0505 | 0.1283 | 0.8501 | 0.0595 | 0.1227 |
|  | 0–2 yr | 0.7620 | 0.0530 | 0.1007 | 0.8138 | **0.0543** | 0.1133 |
|  | 2–8 yr | 0.7503 | 0.0440 | **0.1200** | 0.7939 | 0.0500 | 0.1162 |
|  | 8–18 yr | **0.7980** | **0.0533** | 0.0964 | **0.8642** | 0.0510 | **0.1197** |

Abbreviations: AUROC, area under the receiver operating characteristic curve; AUPRC, area under the precision-recall curve.
