# Supplementary material for: Hypoxemia prediction in pediatric patients under general anesthesia using machine learning: A retrospective observational study and external validation
Source: PLoS One. 2026 Jan 8;21(1):e0339276. doi: 10.1371/journal.pone.0339276 (PMC12782441; doi:10.1371/journal.pone.0339276)
Supplement: S6 Table — This table presents the performance of the XGBoost model for hypoxemia prediction in pediatric patients under general anesthesia, highlighting how different observation window lengths (from 10 to 60 seconds) impact the AUROC, AUPRC, and F1 scores across internal and external validation datasets. Abbreviations: AUROC, area under the receiver operating characteristic curve; AUPRC, area under the precision-recall curve. (DOCX) [file pone.0339276.s006.docx]

S6 Table. Comparative performance of the XGBoost model for hypoxemia prediction in pediatric patients based on changes in observation window length. This table presents the performance of the XGBoost model for hypoxemia prediction in pediatric patients under general anesthesia, highlighting how different observation window lengths (from 10 to 60 seconds) impact the AUROC, AUPRC, and F1 scores across internal and external validation datasets.

| Observational window (sec) | Internal validation | | | External validation | | |
| --- | --- | --- | --- | --- | --- | --- |
|  | AUROC | AUPRC | F1 score | AUROC | AUPRC | F1 score |
| 10 | 0.8363 | 0.1439 | 0.2241 | 0.7578 | 0.0320 | 0.0663 |
| 20 | 0.8408 | 0.1640 | 0.2256 | 0.7685 | 0.0342 | 0.0716 |
| 30 | 0.8468 | 0.1690 | 0.2314 | 0.7767 | 0.0360 | 0.0765 |
| 40 | 0.8479 | 0.1740 | 0.2375 | 0.7809 | 0.0372 | 0.0805 |
| 50 | 0.8486 | 0.1741 | 0.2311 | 0.7812 | 0.0381 | 0.0794 |
| 60 | **0.8550** | **0.1816** | **0.2382** | **0.7857** | **0.0402** | **0.0824** |

Abbreviations: AUROC, area under the receiver operating characteristic curve; AUPRC, area under the precision-recall curve.
