# Supplementary material for: Hypoxemia prediction in pediatric patients under general anesthesia using machine learning: A retrospective observational study and external validation
Source: PLoS One. 2026 Jan 8;21(1):e0339276. doi: 10.1371/journal.pone.0339276 (PMC12782441; doi:10.1371/journal.pone.0339276)
Supplement: S7 Table — In this analysis, the three waveforms—Photoplethysmography, Airway Pressure, and Capnography—were converted into 2D spectrograms, and features were extracted using EfficientNet. These waveform features were then concatenated with features derived from traditional single measurements, which had been extracted using InceptionTime, to be used for the final prediction. Abbreviations: AUROC, area under the receiver operating characteristic curve; AUPRC, area under the precision-recall curve. (DOCX) [file pone.0339276.s007.docx]

S7 Table. Comparative performance of the machine learning model for hypoxemia prediction in pediatric patients using waveform biosignals. In this analysis, the three waveforms—Photoplethysmography, Airway Pressure, and Capnography—were converted into 2D spectrograms, and features were extracted using EfficientNet. These waveform features were then concatenated with features derived from traditional single measurements, which had been extracted using InceptionTime, to be used for the final prediction.

| Pretrained | Internal validation | | | External validation | | |
| --- | --- | --- | --- | --- | --- | --- |
|  | AUROC | AUPRC | F1 score | AUROC | AUPRC | F1 score |
| O | 0.8073 | 0.0936 | 0.0217 | 0.7523 | 0.0497 | 0.0318 |
| X | 0.7723 | 0.0554 | 0.0217 | 0.7395 | 0.0523 | 0.0318 |

Abbreviations: AUROC, area under the receiver operating characteristic curve; AUPRC, area under the precision-recall curve.
