# Supplementary material for: Hypoxemia prediction in pediatric patients under general anesthesia using machine learning: A retrospective observational study and external validation
Source: PLoS One. 2026 Jan 8;21(1):e0339276. doi: 10.1371/journal.pone.0339276 (PMC12782441; doi:10.1371/journal.pone.0339276)
Supplement: S8 Table — This table presents the performance of the four models when trained and evaluated using only the time-series of peripheral oxygen saturation (SpO2) as an input feature. These results serve as a baseline to assess the added predictive value of the multi-variable model. Abbreviations: LSTM, long short-term memory; AUROC, area under the receiver operating characteristic curve; AUPRC, area under the precision-recall curve. (DOCX) [file pone.0339276.s008.docx]

S8 Table. Comparative performance of the machine learning model for hypoxemia prediction in pediatric patients using only the SpO_2_ feature. This table presents the performance of the four models when trained and evaluated using only the time-series of peripheral oxygen saturation (SpO_2_) as an input feature. These results serve as a baseline to assess the added predictive value of the multi-variable model.

|  | Internal validation | | | External validation | | |
| --- | --- | --- | --- | --- | --- | --- |
|  | AUROC | AUPRC | F1 score | AUROC | AUPRC | F1 score |
| XGBoost | **0.7655** | **0.0423** | **0.1029** | 0.7172 | 0.0293 | 0.0592 |
| LSTM | 0.7067 | 0.0309 | 0.0812 | 0.7481 | 0.0375 | 0.0873 |
| Transformer | 0.7189 | 0.0296 | 0.0715 | **0.7730** | 0.0408 | 0.0917 |
| InceptionTime | 0.7261 | 0.0297 | 0.0704 | 0.7613 | **0.0412** | **0.0932** |

Abbreviations: LSTM, long short-term memory; AUROC, area under the receiver operating characteristic curve; AUPRC, area under the precision-recall curve
