# Supplementary material for: Hypoxemia prediction in pediatric patients under general anesthesia using machine learning: A retrospective observational study and external validation
Source: PLoS One. 2026 Jan 8;21(1):e0339276. doi: 10.1371/journal.pone.0339276 (PMC12782441; doi:10.1371/journal.pone.0339276)
Supplement: Attachment — Submitted filename: Response to Reviewers_20250917.docx [file pone.0339276.s010.docx]

PONE-D-25-28107

**Reviewer #1:** This study addresses pediatric hypoxemia prediction using machine learning models. While technically sound, several methodological issues must be clear up before publication.

1. Circular Logic in SpO2-based Prediction
The study defines hypoxemia as SpO2 < 95% while simultaneously using SpO2 as the primary predictor variable. SHAP analysis confirms SpO2 as the most important feature, creating a circular prediction framework. To demonstrate true clinical value, the authors should compare performance against: (1) SpO2-only models, (2) simple threshold-based rules, and (3) existing clinical practice standards if possible. Or state it on limitation.

| **Response** | We thank the reviewer for this insightful comment. We fully agree that addressing the potential for circular reasoning when using SpO_2_ as a primary predictor is crucial to demonstrating the true clinical value of our model. In line with your valuable suggestion, we have conducted an additional experiment comparing our model's performance against a model trained using only the SpO_2_ feature. The results of this new analysis have been added to the manuscript as **S8 Table.**  First, our results show that the model trained solely on SpO_2_ time-series data exhibited significantly lower performance compared to our proposed multi-variable model. This suggests that incorporating other physiological variables causally related to hypoxemia, such as FiO_2_ and EtCO_2_, is critical for enhancing predictive performance. Furthermore, regarding the suggestion of a simple threshold-based rule, we believe such an approach is impractical for the pediatric population. Physiological normal ranges vary dramatically with age and weight, making it infeasible to establish a single, fixed threshold applicable to all patients. In contrast, an intrinsic advantage of our multi-variable model is its ability to learn these complex, non-linear relationships from the data, thereby enabling personalized predictions.  In conclusion, as supported by our SHAP analysis, our model learns the underlying physiological mechanisms of hypoxemia by leveraging the contributions of other variables, rather than merely extrapolating SpO_2_ trends. This characteristic provides higher predictive reliability and clinical explainability than an SpO_2_-only model or a simple threshold rule. We have added a detailed discussion of these findings to the Results section of our manuscript in response to your valuable comment. |
| --- | --- |
| **Revised Manuscript** | Finally, to address the potential for circular reasoning from using SpO_2_ to predict a SpO_2_-defined event, we evaluated the performance of models trained using only SpO_2_ time-series data. The results, detailed inc S8 Table, showed that the SpO_2_-only models had lower performance across most metrics compared to our proposed multi-variable models. For instance, the AUROC of the multi-variable XGBoost model on the internal validation set was 0.855, significantly higher than the 0.766 achieved by its SpO_2_-only counterpart. This demonstrates that incorporating additional physiological variables provides critical contextual information that enhances the model's predictive power beyond simple SpO_2_ trend extrapolation. |

2. Poor Precision Performance
The AUPRC values are critically low: XGBoost achieved 0.1816 (internal) and 0.0402 (external), while LSTM achieved 0.0605(external). The F1 scores remain poor (XGBoost: 0.2382 internal, 0.0824 external). These metrics indicate severely limited precision in clinical application, yet the authors fail to adequately discuss whether such performance justifies implementation or potential harm from false alarms.

| **Response** | We sincerely thank the reviewer for this critical comment. We fully agree that the modest precision of our model warrants a more in-depth discussion regarding its clinical application, and we have approached this issue from the perspective of patient safety as the highest priority.  In predicting a potentially catastrophic event like pediatric hypoxemia, the clinical imperative is unequivocally to minimize false negatives (missed events), even at the cost of accepting some false positives (false alarms). A missed hypoxemic event can lead to severe patient harm, whereas a false alarm results in a transient increase in clinical vigilance and quick patient reassessment. The current performance of our model reflects this necessary clinical trade-off, prioritizing high sensitivity for patient safety.  Notably, we wish to emphasize that the discrepancy between a high AUROC and a modest AUPRC is not unique to our study. The work by Park et al. [13], which we cite in our introduction, also reported a very high AUROC of 0.939 using similar high-frequency data from the same institution (SNUH), yet their AUPRC was similarly low. This suggests that achieving high precision is an inherent challenge for this specific prediction task, likely due to the severe class imbalance and the complexity of the signals.  Therefore, we propose that the value of our model is not as a definitive diagnostic alarm but as a situational awareness support tool for the anesthesiologist. The model provides a "cognitive cue" of increased hypoxemia risk, offering clinicians an opportunity to preemptively reassess the patient and take preventive action. We argue that this is a clinically superior scenario to being caught unaware by a sudden critical event. We have incorporated this detailed discussion into the Limitations section of our manuscript. |
| --- | --- |
| **Revised Manuscript** | This study has several limitations. The study is limited by biases inherent in its retrospective analysis, insufficient evidence linking hypoxemia prediction in children to reduced event occurrences, and the exclusion of high-risk groups, which restricts the applicability of the results. Furthermore, while our models demonstrated predictive capability significantly better than baseline, their precision, as measured by AUPRC, remains modest. This indicates a high rate of false alarms, which could lead to alarm fatigue. Notably, this discrepancy between high AUROC and modest AUPRC is consistent with previous findings on a similar high-frequency pediatric dataset[13], suggesting it may be an inherent challenge of this specific prediction task. However, this reflects a necessary trade-off in a clinical context where failing to predict a true hypoxemic event (a false negative) carries significantly greater risk than a false alarm. This trade-off solidifies the model's current role as a situational awareness support tool rather than a definitive diagnostic alarm.  The model primarily focuses on ventilation-related measures, overlooking circulatory factors that also contribute to hypoxemia. |

3. Inadequate Prediction Timeline Justification
The authors should justify why they defined a 1-minute prediction window. Is this timeframe sufficient for clinical intervention or prevention?

| **Response** | Thank you for this important question regarding the justification for our prediction timeline. We believe that a 1-minute prediction window represents a rational balance between clinical actionability and predictive accuracy, based on the following reasoning.  First, this timeframe accounts for the urgent nature of pediatric hypoxemic events. As our data confirms, once hypoxemia begins, it is a time-critical event that is often resolved within tens of seconds. Given this clinical reality, a 1-minute advance warning provides anesthesiologists with a crucial opportunity to shift from a reactive to a proactive response. A 1-minute window is sufficient for a clinician to recognize an alert and perform key initial preventive actions, such as checking the endotracheal tube position, inspecting the breathing circuit, or adjusting the inspired oxygen concentration.  Second, our methodological choice is consistent with prior research in this field. For instance, the study by Park et al. [13], which we cite, also successfully applied an identical 1-minute prediction window when predicting hypoxemia using similar high-resolution pediatric data. Therefore, we contend that the 1-minute timeframe is a valid and well-founded standard, considering both clinical realities and academic precedent. We have clarified this justification in the Methods section of the revised manuscript. |
| --- | --- |
| **Revised Manuscript** | Each segment for model training was set to 1 minute in length, with segments designated as hypoxemia-predictive intervals if a hypoxemia event occurred within 1 minute after the segment's end.  This 1-minute prediction window was selected as it provides a clinically actionable timeframe for preventive interventions and is consistent with methodologies used in prior studies on pediatric hypoxemia prediction [13]. All other segments were labeled as non-hypoxemia intervals (Fig 2). |

**Reviewer #2:** Many thanks for providing the manuscript for review. The authors utilise physiological data captured from intra-operative anaesthesia devices in order to predict the onset of hypoxemia in a subset of paediatric patients undergoing general anaesthesia in the Republic of Korea. They utilise a rolling window method in which 120 second segments of data are classified as either hypoxemia or no hypoxemia and make use of the first half (60 seconds) to predict occurrence of any hypoxemia in the second (61-120 seconds) half of the window.

The models utilise include XGBoost, LSTM, transformer model and a CNN approach (InceptionTime). The authors report a high AUROC performance for each of the models and utilise this to perform external validation on patients from another institution.

1. I would like to see the Tripod AI checklist included within the supplementary material provided.

► Tripod AI Checklist (https://www.bmj.com/content/bmj/suppl/2024/04/17/bmj-2023-078378.DC5/colg078378.wt2.pdf)

| **Response** | Thank you for your thorough review and valuable suggestion. As requested, we have now included the TRIPOD-AI checklist as **S1 Table**. |
| --- | --- |

2. It would be useful to have further clarity in the introduction on the relationship between a low SpO2 reading and true hypoxemia – what criteria is used to define hypoxemia conventionally e.g. will this include low arterial blood gas oxygen pressures? Helpful for readers who do not work within anaesthesia for example.

| **Response** | Thank you for this excellent suggestion. We agree that clarifying the clinical context of SpO_2_ and its relationship to true hypoxemia is very helpful for a broader audience.  Conventionally, the gold standard for diagnosing hypoxemia is a low partial pressure of arterial oxygen (PaO_2_) measured directly from an arterial blood gas analysis. However, obtaining an arterial blood gas is invasive and time-consuming, making it unsuitable for continuous, real-time monitoring during surgery where rapid changes can occur.  For this reason, clinical practice relies on peripheral oxygen saturation (SpO_2_) from pulse oximetry as a non-invasive, continuous surrogate for detecting hypoxemia. While SpO_2_ generally reflects the true arterial oxygen saturation (SaO_2_) well, it can be susceptible to artifacts from patient movement, poor peripheral circulation, or incorrect sensor placement, leading to "false hypoxemia." To mitigate this and enhance the clinical validity of our data, all hypoxemic events in our study were labeled by two expert anesthesiologists who comprehensively reviewed not only the SpO_2_ values but also related biosignals, ventilator parameters, and waveforms.  We have now added this background information to the Introduction section of our manuscript to ensure that readers can more clearly understand the clinical context and terminology of our study. |
| --- | --- |
| **Revised Manuscript** | However, false hypoxemia can complicate monitoring in pediatric patients, increase the workload of anesthesiologists, and obstruct careful monitoring efforts. A reliable study using Anesthesia Information Management Systems found that the incidence of pediatric intraoperative hypoxemia was 12%, with only 54% classified as true hypoxemia[2]. Conventionally, true hypoxemia is defined by a low arterial oxygen pressure (PaO_2_) from arterial blood gas analysis. Relying solely on SpO_2_ readings during hypoxemia events is insufficient for accurately assessing the condition of the patient. Thus, immediate assessment of hypoxemia requires evaluating various other patient-specific parameters. Additionally, parameters such as airway pressure and end-tidal carbon dioxide (EtCO_2_) waveform, along with related metrics, influence intuitive interpretation during monitoring[6,7]. Therefore, clinicians rely on peripheral oxygen saturation (SpO_2_) as a real-time surrogate, although it can be prone to artifacts. |

3. I would like to also see data on the different types of surgery cases included in the baseline descriptions to understand the population better.

| **Response** | Thank you for your valuable suggestion to clarify the characteristics of our study population. We agree that understanding the influence of surgical type is important.  While further analysis of surgical types is possible for the external validation dataset (CNUH), we would like to emphasize that we sought to control for heterogeneity arising from surgical type during the study design phase. As detailed in our Methods section, we deliberately excluded specific procedures where the mechanism or incidence of hypoxemia is expected to differ significantly from general cases (e.g., cardiac surgery, pulmonary surgery, and airway surgeries that induce apnea). We would also like to note that for the training and internal validation dataset (SNUH), we were unable to retrospectively obtain the same data due to institutional restrictions following a rigorous de-identification protocol.  This design ensures that our study cohort is representative of a more general pediatric surgical population rather than being skewed by specific high-risk procedures. Therefore, we believe the key takeaway is that our model focuses on predicting hypoxemia in the context of general anesthetic management, rather than being dependent on the distribution of specific surgical types. To clarify this point, we have added a sentence to the Methods section explaining this design intent. |
| --- | --- |
| **Revised Manuscript** | Patients without Vital Recorder data during anesthesia, including patients undergoing cardiac or pulmonary surgery, those who underwent procedures that induce apnea (such as airway surgeries), and those with preoperative oxygen saturation <95% were excluded. This exclusion was intended to minimize confounding factors from procedure-specific causes of hypoxemia, thereby focusing the model on predicting events arising from general anesthetic management. Additionally, to exclude clinically insignificant and noisy hypoxemic events, the following exclusion criteria were applied: when the pulse rate measured via pulse oximetry exceeded 20% of the heart rate recorded by an electrocardiogram; when the plethysmography waveform was severely distorted; when an anesthesiologist recorded that the oxygen saturation was inaccurate (Fig 1). |

4. The authors mention that relying on SpO2 readings alone is insufficient for accurately assessing true hypoxemia – however their definition of hypoxemia used for model training and evaluation appears to be based largely on SpO2 alone. Can they elaborate on what processes are used to ensure this is clinically valid (e.g. annotation process)

| **Response** | Thank you for this very important question regarding the clinical validity of our labeling process. Your comment has helped us realize that the efforts we made to overcome the limitations of relying solely on SpO_2_ values may not have been communicated with sufficient clarity in the manuscript. We would like to elaborate on this process.  As you correctly pointed out, we were aware of the risk of "false hypoxemia" that can arise from relying on SpO_2_ values alone. To minimize this, we implemented a multi-faceted validation procedure during the study design phase. First, to exclude artifacts, we filtered out segments where: (1) the pulse rate from oximetry differed by more than 20% from the ECG heart rate, (2) the plethysmography waveform was severely distorted, or (3) an anesthesiologist had manually recorded the SpO_2_ reading as inaccurate. Subsequently, the final labeling process was conducted by two expert anesthesiologists who comprehensively reviewed other relevant biosignals. This was followed by an inter-observer double-check to create a systematic and robust annotation process.  To address the lack of clarity in our original manuscript, we have now revised the Methods section to explicitly state that these efforts were a core part of our methodology, undertaken to ensure the accurate identification of "true hypoxemia." We appreciate you bringing this to our attention. |
| --- | --- |
| **Revised Manuscript** | Data segments of 1-minute duration were used for model training. These segments were generated with an initiation time staggered by 2 seconds from the preceding segment. To ensure that the SpO2-defined labels reflected clinically valid hypoxemic events, all labeling processes were conducted by two expert anesthesiologists and were double-checked by inter-observers; any disagreements during the validation process were resolved through discussion. |

5. What is the nature of the ‘vital signs’ used to train the initial models? Are these tabular data extracted from the machines, continuous waveforms or a composite – useful to be explicit and describe the data, data quality, and what pre-processing work was involved in making them useable.

| **Response** | Thank you for this important question requesting clarification on the nature of our data and the preprocessing pipeline. We agree that these details are essential for reproducibility and have revised the Methods section accordingly.  The data used in our study were not raw continuous waveforms (such as ECG or arterial pressure waves) but rather sequential tabular data, comprising numerical time-series data extracted from the prospective registry of vital signs for surgical patients at 2-second intervals. The six key physiological parameters used for model training were peripheral oxygen saturation (SpO_2_), fraction of inspired oxygen (FiO_2_), end-tidal carbon dioxide (EtCO_2_), peak inspiratory pressure (PIP), tidal volume (TV), and minute ventilation (MV). These time-series features were combined with four static demographic variables (height, weight, sex, and age) to form the complete input for the models. A visual example of these signals is provided in the figure below.    Our preprocessing workflow was as follows. First, any intermittent missing values were imputed with zero. The continuous data stream was then segmented into 60-second (30 time-point) input windows. Finally, to ensure that each signal contributed equally to the model training process despite having different units and ranges, each of the six time-series signals within every input window was independently normalized to a [0, 1] range using min-max scaling. This is a crucial step for the stable training of deep learning models.  We have added these detailed descriptions to a new subsection titled “Preprocessing" in the Methods section of our revised manuscript to enhance the clarity and reproducibility of our work. |
| --- | --- |
| **Revised Manuscript** | The vital signs used for model input were sequential tabular data, comprising numerical time-series data sampled at 2-second intervals. The six key physiological parameters used were SpO2, FiO2, EtCO2, PIP, TV, and MV.  These time-series features were combined with four static demographic variables (height, weight, sex, and age) to form the complete input for the model. To prepare these features for the models, we first imputed any missing values with zero. Subsequently, for each 60-second input window, each of the six time-series signals was independently scaled to a range of [0, 1] using local min-max normalization to ensure stable model training. |

**6.** How were the hyperparameters chosen? Is this through cross-validation if so what are the performances? Reporting only the hold out set metrics for model development may not provide the full picture.

| **Response** | Thank you for this important question regarding the rigor of our model development process. To provide a clear answer, we would like to elaborate on the hyperparameter optimization procedure we conducted.  For the LSTM, XGBoost, and Transformer models, we performed a systematic search to identify the optimal combination of key hyperparameters from a predefined grid. This search was conducted on the training set using a 5-fold cross-validation strategy, and the hyperparameter combination that yielded the highest average AUROC was selected as the final configuration for each model. For the InceptionTime model, we adopted the architecture and parameters proposed by the original authors to leverage its proven performance on time-series classification tasks.  To ensure full transparency and reproducibility, we have created a new **S3 Table**, which details the entire hyperparameter search space, the final selected parameters, and other key training parameters (e.g., learning rate, batch size). We have also added a detailed description of this process to the "Machine learning models and hyperparameter optimization" subsection of our manuscript. |
| --- | --- |
|  | This study used four types of machine learning models: XGBoost, LSTM, InceptionTime, and Transformer[9,15-17]. To optimize each model, we performed a hyperparameter search for XGBoost, LSTM, and Transformer using a 5-fold cross-validation strategy on the training set. The parameter combination that yielded the highest average AUROC was selected as the final configuration. For the InceptionTime model, we adopted the architecture from the original publication due to its proven performance on time-series classification tasks. The complete search space and the final selected hyperparameters for all models are detailed in S3 Table. |

7. The model utilises SpO2 as their primary input feature in order to predict subsequent SpO2. How is the nature of repeated measurement accounted for in the models (if they need to be). For example, I would imagine a low SpO2 in the first half of the window is predictive of a low SpO2 in the second half?

| **Response** | Thank you for this insightful question regarding the technical core of our time-series modeling, specifically concerning the autocorrelation of the data. Your comment has given us a welcome opportunity to clarify how our models handle the nature of repeated measurements.  You are correct in your observation that SpO_2_ values exhibit high temporal correlation, meaning a low SpO_2_ in the past is naturally predictive of a low SpO_2_ in the future. While our original explanation may have been insufficient, the LSTM and Transformer models we employed are architecturally designed to intrinsically learn precisely this type of temporal dependency. These models go beyond simple extrapolation of past values; they are built to consider complex temporal interactions, such as trends and variability in the SpO_2_ signal over the observation window, as well as its interplay with other biosignals like FiO_2_ and EtCO_2_.  To empirically verify the added value of this multi-variable approach, we conducted an additional experiment, as you suggested, comparing our models' performance to that of models trained using only the SpO_2_ time-series (results in **S8 Table**). The results confirmed that performance was higher when other physiological variables were included. We cautiously interpret this to mean that our models learn information related to the underlying physiological mechanisms of hypoxemia, moving beyond simple autocorrelation. We have added a discussion of these findings to the "Comparative performance in hypoxemia prediction" subsection of our manuscript. |
| --- | --- |
| **Revised Manuscript** | Finally, to address the potential for circular reasoning from using SpO_2_ to predict a SpO_2_-defined event, we evaluated the performance of models trained using only SpO_2_ time-series data. The results, detailed in S8 Table, showed that the SpO_2_-only models had lower performance across most metrics compared to our proposed multi-variable models. For instance, the AUROC of the multi-variable XGBoost model on the internal validation set was 0.855, significantly higher than the 0.766 achieved by its SpO_2_-only counterpart. This demonstrates that incorporating additional physiological variables provides critical contextual information that enhances the model's predictive power beyond simple SpO_2_ trend extrapolation. |

8. Can the authors comment on why XGBoost was the best performing in the internal validation set but did not perform as well in external validation? There is a disproportionate difference between XGBoost and LSTM approaches internally.

| **Response** | Thank you for this excellent question regarding the reasons for the differing performance of the models on the internal and external validation sets. We consider this phenomenon to be one of the key findings of our study, as it underscores the importance of external validation and reveals how model architecture can influence generalizability.  Our interpretation is as follows. As a tree-based model, XGBoost excels at capturing complex, non-linear relationships and specific data distribution patterns present within the training data (from SNUH). Indeed, as we show in Table 1, the datasets from the two hospitals exhibit statistically significant differences in the frequency and duration of hypoxemic events. XGBoost was likely highly optimized for these unique patterns in the training and internal validation dataset, leading to its superior performance internally. However, its performance degraded on the external validation set, where these specific patterns were not present.  In contrast, the Transformer model, which performed best on the external validation set, uses a self-attention mechanism to learn general rules about which time points in a series are important. This may have been more advantageous for capturing more fundamental and generalizable physiological patterns that persist even when the dataset distribution changes.  In conclusion, this result suggests that a trade-off can exist between optimization for a specific dataset and robust performance on heterogeneous data. It clearly demonstrates that a model's true clinical utility is difficult to assess with single-center data alone. We have incorporated this in-depth discussion into the Discussion section of our manuscript. |
| --- | --- |
| **Revised Manuscript** | However, other research has shown that machine learning approaches, such as the GBM, achieved the best performance for hypoxemia prediction[13]. Our findings align with this, indicating that even machine learning models like XGBoost, which lack a native sequential architecture[24], can achieve high performance by effectively capturing the complex interactions between biosignals and demographic data. Furthermore, as with previous studies, it is crucial to evaluate models using external validation rather than relying solely on internal validation to confirm their generalizability[10-13]. |

9. The dataset is highly imbalanced with only around 1.4% at most positive. This means that the AUROC will be high regardless and therefore can be misleading to present this as the primary result. The AUPRC for the external validation is around 0.06 (where the baseline will be around 0.012) – can the authors comment on the suitability of the model for clinical implementation? Is this a useful model to inform clinicians?

| **Response** | We sincerely thank the reviewer for this critical comment. We fully agree that the modest precision of our model warrants a more in-depth discussion regarding its clinical implementation, and we have approached this issue from the perspective of patient safety as the highest priority.  In predicting a potentially catastrophic event like pediatric hypoxemia, the clinical imperative is unequivocally to minimize false negatives (missed events), even at the cost of accepting some false positives (false alarms). A missed hypoxemic event can lead to severe patient harm, whereas a false alarm results in a transient increase in clinical vigilance and quick patient reassessment. The current performance of our model reflects this necessary clinical trade-off, prioritizing high sensitivity for patient safety.  Notably, we wish to emphasize that the discrepancy between a high AUROC and a modest AUPRC is not unique to our study. The work by Park et al. [13], which we cite, also reported a very high AUROC of 0.939 using similar high-frequency data from the same institution (SNUH), yet their AUPRC was similarly low. This suggests that achieving high precision is an inherent challenge for this specific prediction task, likely due to the severe class imbalance and the complexity of the signals.  Therefore, we propose that the value of our model is not as a definitive diagnostic alarm but as a situational awareness support tool for the anesthesiologist. The model provides a "cognitive cue" of increased hypoxemia risk, offering clinicians an opportunity to preemptively reassess the patient and take preventive action. We argue that this is a clinically superior scenario to being caught unaware by a sudden critical event. We have incorporated this detailed discussion into the Limitations section of our manuscript. |
| --- | --- |
| **Revised Manuscript** | This study has several limitations. The study is limited by biases inherent in its retrospective analysis, insufficient evidence linking hypoxemia prediction in children to reduced event occurrences, and the exclusion of high-risk groups, which restricts the applicability of the results. Furthermore, while our models demonstrated predictive capability significantly better than baseline, their precision, as measured by AUPRC, remains modest. This indicates a high rate of false alarms, which could lead to alarm fatigue. Notably, this discrepancy between high AUROC and modest AUPRC is consistent with previous findings on a similar high-frequency pediatric dataset[13], suggesting it may be an inherent challenge of this specific prediction task. However, this reflects a necessary trade-off in a clinical context where failing to predict a true hypoxemic event (a false negative) carries significantly greater risk than a false alarm. This trade-off solidifies the model's current role as a situational awareness support tool rather than a definitive diagnostic alarm.  The model primarily focuses on ventilation-related measures, overlooking circulatory factors that also contribute to hypoxemia. Incorporating variables such as blood pressure and ECG data could improve the model’s relevance to real-world surgical scenarios. Future research should adopt a prospective approach with a broader patient demographic, include real-time data collection to enhance precision and clinical applicability, and prioritize external validation across multiple institutions to ensure the model’s generalizability and robustness. |

10. I would disagree with the statement that machine learning models are not inherently designed (or suitable) for time series data – when alternatives include linear modelling.

| **Response** | Thank you for your precise comment. We acknowledge that our original phrasing was misleading and agree with you entirely; it is incorrect to state that machine learning models are unsuitable for time-series data, and we apologize for our lack of clarity.  Our intention was to highlight the fundamental architectural differences in how various models process temporal information, specifically between tree-based models like XGBoost and recurrent/attention-based models like LSTM/Transformer. Whereas models like LSTM are explicitly designed to model the sequential dependency of data, XGBoost treats time-series data as a fixed-length feature vector, excelling at capturing complex, non-linear interactions between variables. The strong performance of XGBoost in our study was an interesting finding, as it demonstrated that significant predictive patterns could be learned even with the latter approach.  We have revised the relevant sentence in the Discussion section to clarify this important nuance, thanks to your valuable feedback. |
| --- | --- |
| **Revised Manuscript** | However, other research has shown that machine learning approaches, such as the GBM, achieved the best performance for hypoxemia prediction[13]. Our findings align with this, indicating that even machine learning models like XGBoost, which lack a native sequential architecture[24], can achieve high performance by effectively capturing the complex interactions between biosignals and demographic data. Furthermore, as with previous studies, it is crucial to evaluate models using external validation rather than relying solely on internal validation to confirm their generalizability[10-13]. |

**11.** What is the significance of the SHAP analysis and figure – especially when height is the greatest contributor to model prediction but most features are centred around 0.

| **Response** | Thank you for your sharp observation and question regarding our SHAP analysis. We agree that the high overall impact of height is an interesting finding, and we also consider it an important clue that supports the clinical validity of our model.  In pediatric patients, height serves as a critical proxy variable that is intricately linked to age, weight, lung volume, and physiological reserve. Therefore, the high importance assigned to height by the model signifies that it has successfully learned the complex, non-linear relationship between these composite patient characteristics and the risk of hypoxemia. The wide distribution of its SHAP values around zero suggests that the influence of height is context-dependent, increasing or decreasing risk based on its interaction with other variables.  However, the most crucial significance of this SHAP analysis, we believe, is that in addition to this complex influence of demographic variables, the model correctly distinguishes the importance of key physiological variables in a manner consistent with clinical intuition. As described in the manuscript, our model identified variables directly related to oxygenation and ventilation (SpO_2_, FiO_2_, EtCO_2_) as more impactful predictors than mechanical ventilation parameters (PIP, TV, MV). This provides strong evidence that while the model adjusts risk based on static patient characteristics (like height and weight), its final predictions are weighted toward the core physiological indicators that best reflect immediate risk. This is precisely what makes the model's prediction process trustworthy.  We have revised the "Feature importance analysis" subsection and the caption for Figure 4 to reflect this detailed interpretation. |
| --- | --- |
| **Revised Manuscript** | **Feature importance analysis**  To visually explain the impact of the variables, we used SHAP to illustrate how they influence the model's predictions (Fig. 4).  The demographic information shows wide distribution of SHAP values across both positive and negative domains suggests their influence is highly context-dependent, varying based on interactions with other physiological variables. The SHAP values for SpO₂, FiO₂, and EtCO₂ show a broader distribution with greater density of points indicating both positive and negative effects on the model’s predictions, compared to those for PIP, TV, and MV. This indicates that SpO_2_, FiO_2_, and EtCO_2_ have a more significant impact on the model's predictions. Additionally, the variables PIP, TV, and MV predominantly contributed negatively to the model's predictions, as their distributions are primarily located on the left side of zero. This suggests that higher values of these variables may adversely affect the outcome.  **Fig 4.** SHAP values for feature importance in the model's predictions of intraoperative hypoxemia**.** SHAP values for internal (a) and external validations (b). The plot shows that demographic features have a highly context-dependent influence. In contrast, key physiological variables such as SpO_2_, FiO_2_, and EtCO_2_ exhibit a more significant and direct impact on the model's predictions compared to mechanical ventilation parameters like PIP, TV, and MV, aligning with clinical intuition. SHAP, Shapley Additive exPlanations; SpO_2_, peripheral oxygen saturation; FiO_2_, fraction of inspired oxygen; EtCO_2_, end-tidal carbon dioxide; PIP, peak inspiratory pressure; TV, tidal volume and MV, minute ventilation. |

12. Could the captions and figures be provided in higher resolution?

| **Response** | We appreciate this valuable feedback. In accordance with your request, Figures 3 and 4 have been replaced with high-resolution versions. |
| --- | --- |

13. Can I urge the anonymised data to be provided as part of submission given this would be valuable contribution to medical research into signal analysis where possible.

| **Response** | We strongly agree with the reviewer regarding the academic value and importance of data sharing for reproducibility. Our research team is also committed to the principles of transparency and data sharing in scientific discovery.  However, the data used in this study involve sensitive medical information from pediatric patients. The scope of our initial Institutional Review Board (IRB) approval was limited to internal research use and did not include provisions for public data release. Due to stringent ethical and legal regulations protecting patient confidentiality, we are currently unable to deposit the dataset in a public repository. We appreciate your understanding in this matter.  Nevertheless, we highly value collaboration with other researchers. Therefore, we have established a formal procedure for data sharing for legitimate research purposes. Interested researchers may contact the corresponding author with a research proposal, and we are committed to actively supporting and facilitating the process for obtaining the necessary approvals from the respective IRBs and Data Review Boards (DRBs) of the relevant institutions (Chungnam National University Hospital and Seoul National University Hospital). We believe this controlled access approach allows us to contribute to the scientific community while rigorously protecting patient confidentiality. This process has been clearly described in the manuscript's Data Availability Statement.  Additionally, we would like to take this opportunity to introduce a valuable public database that may be of great interest to you and other researchers: **VitalDB (**[https://vitaldb.net/dataset/](https://www.google.com/url?sa=E&q=https%3A%2F%2Fvitaldb.net%2Fdataset%2F)**)**. VitalDB is a large-scale, publicly available dataset established by Seoul National University Hospital. It contains high-resolution biosignals (e.g., 500 Hz waveforms) and over 60 clinical variables from 6,388 surgical patients. This dataset is provided free of charge to facilitate research and development of new medical AI algorithms using intraoperative monitoring signals. While access to our specific pediatric dataset is restricted, we believe that VitalDB could serve as an excellent and valuable resource for research in signal analysis. |
| --- | --- |

**[Other Minor Revisions]**

Finally, in the course of preparing this revision, we conducted a thorough final review of the entire manuscript. During this process, we identified a minor error in the "Implementation details" subsection. We had incorrectly stated that model weights were selected based on the best AUPRC, when in fact they were selected based on the best **AUROC**, consistent with our hyperparameter tuning strategy. We sincerely apologize for this oversight and have corrected this statement in the revised manuscript to ensure its complete accuracy.

We thank you once again for your time and invaluable contributions to improving our manuscript.

**[Revised Manuscript]**

**Implementation details**

All models were trained for 100 epochs using five-fold cross-validation to improve robustness, with binary cross-entropy loss as the optimization criterion. As hypoxemic events were nearly 100 times rarer than non-hypoxemic periods, the loss function for class 1 was heavily weighted compared with class 0 to address the data imbalance issue.

The batch size was set to 1,024, and all deep learning models used the Adam optimizer with an initial learning rate of 0.001, beta1 of 0.9, and beta2 of 0.999. The model weights that achieved the best area under the receiver operating characteristic curve (AUROC) across all epochs were selected for evaluation. The F1 score was calculated, and the optimal threshold for binary classification was chosen based on the highest F1 score.
